# Role of phosphate limitation and pyruvate decarboxylase in rewiring of the metabolic network for increasing flux towards isoprenoid pathway in a TATA binding protein mutant of *Saccharomyces cerevisiae*

**DOI:** 10.1186/s12934-018-1000-1

**Published:** 2018-09-21

**Authors:** Manisha Wadhwa, Sumana Srinivasan, Anand K. Bachhawat, K. V. Venkatesh

**Affiliations:** 1 Department of Biological Sciences, Indian Institute of Science Education and Research, Mohali, Mohali, India; 2 Department of Chemical Engineering, Indian Institute of Technology, Bombay, Mumbai, India

**Keywords:** *SPT15*, Metabolic flux distribution, Phosphate, NADPH, Isoprenoid pathway, *PDC6*

## Abstract

**Background:**

Production of isoprenoids, a large and diverse class of commercially important chemicals, can be achieved through engineering metabolism in microorganisms. Several attempts have been made to reroute metabolic flux towards isoprenoid pathway in yeast. Most approaches have focused on the core isoprenoid pathway as well as on meeting the increased precursors and cofactor requirements. To identify unexplored genetic targets that positively influence the isoprenoid pathway activity, a carotenoid based genetic screen was previously developed and three novel mutants of a global TATA binding protein SPT15 was isolated for heightened isoprenoid flux in *Saccharomyces cerevisiae*.

**Results:**

In this study, we investigated how one of the three *spt15* mutants, *spt15_Ala101Thr*, was leading to enhanced isoprenoid pathway flux in *S. cerevisiae*. Metabolic flux analysis of the *spt15_Ala101Thr* mutant initially revealed a rerouting of the central carbon metabolism for the production of the precursor acetyl-CoA through activation of pyruvate-acetaldehyde-acetate cycle in the cytoplasm due to high flux in the reaction caused by pyruvate decarboxylase (*PDC*). This led to alternate routes of cytosolic NADPH generation, increased mitochondrial ATP production and phosphate demand in the mutant strain. Comparison of the transcriptomics of the *spt15_Ala101Thr* mutant cell with *SPT15*WT bearing cells shows upregulation of phosphate mobilization genes and pyruvate decarboxylase 6 (*PDC6*). Increasing the extracellular phosphate led to an increase in the growth rate and biomass but diverted flux away from the isoprenoid pathway. *PDC6* is also shown to play a critical role in isoprenoid pathway flux under phosphate limitation conditions.

**Conclusion:**

The study not only proposes a probable mechanism as to how a *spt15_Ala101Thr* mutant (a global TATA binding protein mutant) could increase flux towards the isoprenoid pathway, but also *PDC* as a new route of metabolic manipulation for increasing the isoprenoid flux in yeast.

**Electronic supplementary material:**

The online version of this article (10.1186/s12934-018-1000-1) contains supplementary material, which is available to authorized users.

## Background

Isoprenoids/terpenoids are a large family of compounds, many of which are commercially important molecules. A large amount of effort has been focused on engineering microbes such as *Escherichia coli* and *Saccharomyces cerevisiae* for their commercial production. In yeasts, the isoprenoid/mevalonic acid (MVA) biosynthetic pathway has been engineered to increase the metabolic flux and the yield of terpenoids. These include overexpression of cytosolic truncated HMG-CoA reductase (*tHMG1*) (to remove feedback regulation) [1], down regulation of squalene synthase (*ERG9*) (to reduce branching off pathway) [2] and increasing the expression of FPP synthase (*ERG20*) [3]. Most attempts have focused almost exclusively on the genes and regulatory controls of the isoprenoid pathway. Some efforts have also been directed towards increasing the cytosolic pools of the precursor acetyl-CoA by engineering pyruvate dehydrogenase (PDH) bypass [4], overexpression of alcohol dehydrogenase (*ADH2*) for increasing acetaldehyde pools, a precursor for acetyl-CoA through production from ethanol [5], decreasing competition for cytosolic acetyl-CoA in the glyoxylate cycle with malate synthase (*MLS1*) and citrate synthase (*CIT2*) gene deletions [6], and exogenous addition of acetate in the exponential phase [7]. As increasing acetyl-CoA pools from acetate by acetyl-CoA synthases (*ACS1*, *ACS2*) requires ATP, many metabolic engineering strategies have tried to reduce overall ATP cost by decoupling acetyl-CoA supply from ATP hydrolysis [8–11]. Further, overexpression of glucose-6-phosphate dehydrogenase (*ZWF1*), NADH kinase (*POS5*) genes to increase NADPH pools, the cofactor required for isoprenoid pathway has been attempted [12]. However, we ask, apart from the core pathway and the precursors that influence the isoprenoid pathway, are there any hitherto unexplored factors that have a positive influence over the isoprenoid flux and yields? We recently described a genetic screen to isolate mutants increasing the isoprenoid flux with a goal to identify unexplored genes or mutants [13]. Using this approach we were able to isolate three novel mutants of *SPT15*, a TATA-binding protein (TBP) that is an essential general transcription factor required for all three RNA polymerases of yeast. These *spt15* mutant yeast strains carry a native copy of *SPT15* gene in the genome and one additional mutant copy of *spt15* in the plasmid. In a previous study [14], different *spt15* mutants were isolated for phenotypic improvement such as stress tolerance by global transcription machinery engineering (gTME) (Additional file 1: Table S1). Since *spt15* mutants cause pleiotropic changes in gene transcription, the mechanism by which these mutants improve phenotype remains elusive. Similarly, it is not clear as to how the novel *spt15* mutants increased the isoprenoid flux.

In the present study, we have attempted to understand the basis for increased isoprenoid flux of one of three mutants, *spt15_Ala101Thr*. This mutant was isolated using a yeast based genetic screen for isoprenoid flux [13]. This *spt15_Ala101Thr* mutant strain was shown to have the ability to increase the yield of not only the heterologously produced tetraterpenoids (carotenoids) in *S. cerevisiae* but also the yield of heterologously produced sesquiterpenes (α-Farnesene) in *S. cerevisiae* showing that they had the capacity of increasing the general isoprenoid flux [13]. Using a combination of metabolic flux analysis, transcriptomics and validation by phenotypic evaluation, we demonstrate how the *spt15_Ala101Thr* mutation causes rewiring of the metabolic network for increasing the metabolic flux towards the isoprenoid pathway. In addition to yielding insights into the mechanism for increased isoprenoid pathway flux in the *spt15_Ala101Thr* mutant, this study also proposes new routes of metabolic manipulation for increasing the isoprenoid flux in yeast.

## Results

### Metabolic flux balance analysis of control and mutant strain with whole genome scale metabolic model reveals carbon rerouting in mutant strain for increasing the isoprenoid flux

*Saccharomyces cerevisiae* BY4741 strain containing a native copy of *SPT15* in the genome was used as a wild type strain. Growth experiments were performed in order to evaluate the fluxes in the two strains; control 101a (transformed with *SPT15*WT in wild type strain) and mutant 102a (transformed with *spt15_Ala101Thr* mutant in wild type strain) (Additional file 1: Table S2). It was observed that the mutant 102a strain, had a low growth rate (0.19 h^−1^) as compared to the wild type/control 101a strain (0.24 h^−1^) as shown in Additional file 1: Fig. S1. Further, a 2.25 fold increase in the isoprenoid pathway flux was also observed in the 102a mutant strain, evaluated through carotenoid accumulation in the genetic screen [13].

**Fig. 1:**
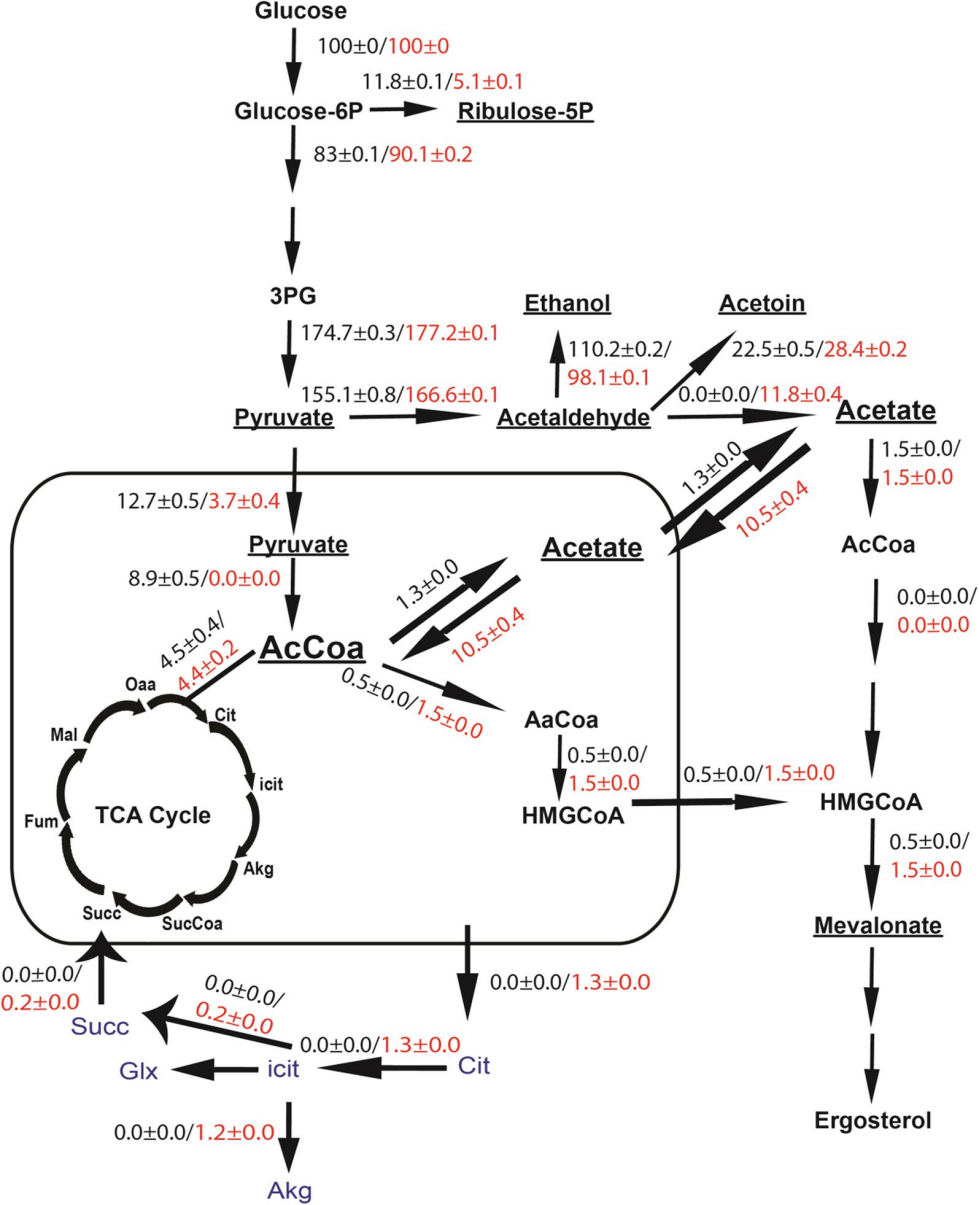
Flux distribution in central metabolism of control 101a (*SPT15*WT) and mutant 102a (*spt15_Ala101Thr*) strain without transcriptomic changes. Fluxes are simulated with iMM904 model using experimentally determined constraints for *S. cerevisiae* strain carrying either *SPT15*WT plasmid (Control 101a strain) or *spt15_Ala101Thr* plasmid (mutant 102a strain). Fluxes in wild type and mutant are shown in black and red colours. The metabolites showing major changes are underlined. Glyoxylate cycle which is present only in mutant strain is shown in blue colour

The detailed flux balance distribution in the central carbon metabolism of 101a and 102a strains was determined (Additional file 1: Fig. S2) using the constraints shown in Additional file 1: Table S3A. A simplified flux balance distribution in central carbon metabolism for comparison of 101a (wild type) and 102a (mutant) strain is shown in Fig. 1. The phenotypic state characterized by the fluxes in the metabolic network was used to postulate mechanisms for increased isoprenoid pathway flux. The mutant strain demonstrated a 50% lower flux through PPP which correlated with the lower growth rate (Fig. 1). Most of the pyruvate formed via glycolysis was channeled towards acetaldehyde (via pyruvate decarboxylase, *PDC*) in the cytosol (~ 89%) and about 7% was transported to mitochondria in control 101a strain, while these percentages were 94% and 2% in the mutant 102a strain (Fig. 1). The excess carbon flux from pyruvate towards acetaldehyde (via *PDC*) in the mutant increased the flux through acetoin and acetate in the cytosol, resulting in 11% lower ethanol formation. The excess acetate in the mutant was shunted across the mitochondria towards mitochondrial acetyl-CoA pools and a threefold increase of flux towards isoprenoid pathway through HMG-CoA (Fig. 1). It should be noted that in the control 101a strain, the flux from acetaldehyde was only towards acetoin. The acetate was formed mainly through acetyl-CoA (formed from pyruvate via PDHC) and acetyl CoA was channeled towards TCA cycle, thereby reducing the flux in the isoprenoid pathway through HMG-CoA (Fig. 1).

The flux balance analysis indicated an increase in mitochondrial ATP (~ 29%) in the mutant strain (Additional file 1: Table S4). Increase in the demand of mitochondrial ATP was due to the flux in the reactions catalyzed by acetyl-CoA synthetase and adenylate kinase. Owing to increase in the demand for ATP synthesis by mitochondrial ATP synthase, the cytosolic inorganic phosphate levels were increased as observed in the high flux of inorganic di-phosphatases (Additional file 1: Table S5). In addition, the transport of phosphate from the cytosol to the mitochondria increased due to increase in flux in the reaction of mitochondrial phosphate transporter by 3% (Additional file 1: Table S6). Therefore in the 102a mutant strain, we can postulate that phosphate was imported from cytosol to the mitochondria to increase ATP production.

Therefore based on the metabolic flux simulation results, we can summarize that the *spt15_Ala101Thr* mutation caused rewiring of carbon metabolism so as to increase precursor pools, acetyl CoA to increase the isoprenoid pathway flux and this was also accompanied by a higher inorganic phosphate demand in the cell.

### Transcriptome profile analysis of mutant strain indicates upregulation of phosphate pathway and pyruvate decarboxylase

**Fig. 2:**
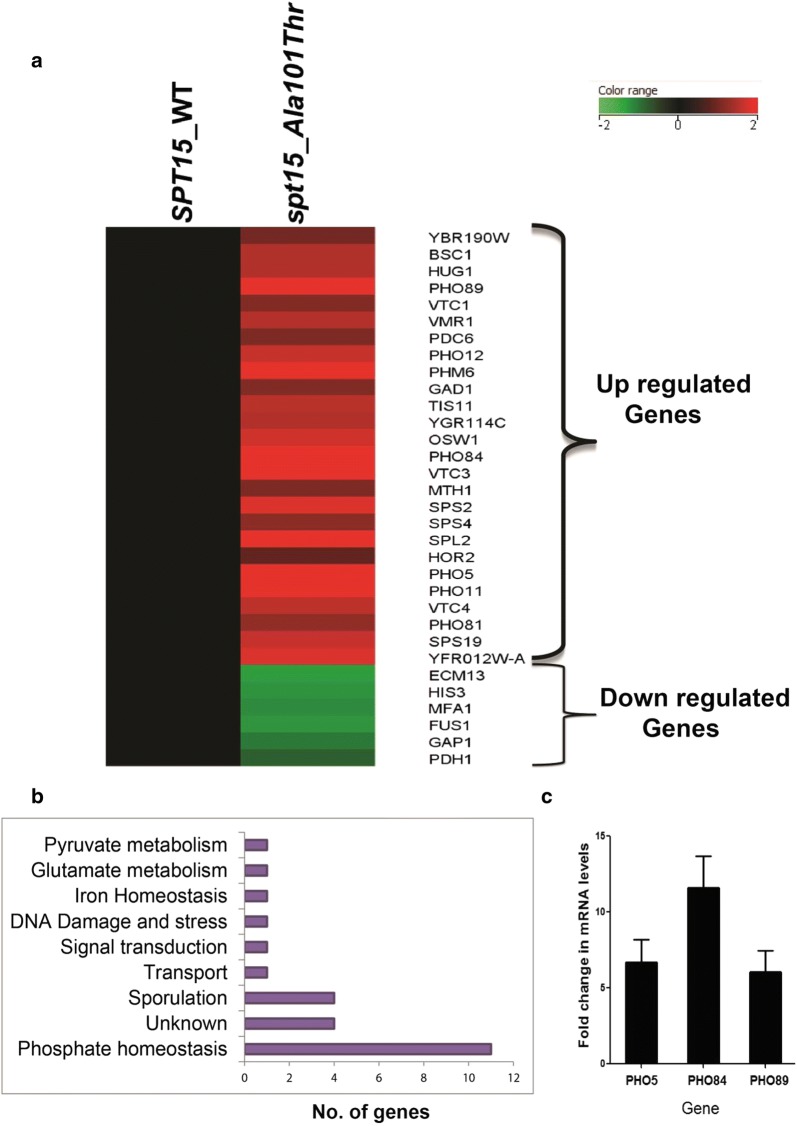
Microarray data analysis of 101a (*SPT15*WT) and 102a (*spt15_Ala101Thr*) strains of *S. cerevisiae.* **a** Heat map showing differentially expressed genes in *spt15_Ala101Thr* strain **b** Bar chart showing number of genes up regulated in different pathways **c** Validation of top up regulated transcripts- *PHO5*, *PHO84* and *PHO89* of microarray by qPCR. For qPCR, three independent experiments with biological duplicates were performed and data is shown from one independent experiment

In order to validate the predicted mechanism for increased isoprenoid flux in mutant 102a strain, we carried out transcriptomic analysis of the mutant 102a and control 101a strain and differential expression of genes with fold change greater than or equal to 2 (with p < 0.05) were tabulated. We observed a differential expression of 30 genes, out of which 25 genes were upregulated and only 5 genes were downregulated in the mutant 102a strain as compared to control 101a strain (Fig. 2a). We also included two other metabolic genes corresponding to enzymes, *HOR2* (glycerol-3-phosphatase) and *PDH1* (2-methyl citrate dehydratase), with *p* value < 0.05 and fold change 1.86 and 0.56 respectively. Interestingly, out of the 25 upregulated genes, 11 genes were from the phosphate regulon (PHO), with a greater than threefold upregulation (Fig. 2b). The upregulation of three PHO regulon genes, namely, *PHO5*, *PHO84* and *PHO89,* were validated using q-PCR (Fig. 2c). Besides the PHO regulon genes, there was upregulation of glycerol phosphatase (*HOR2*) which hydrolyzes the phosphate ester bond of glycerol phosphate, found in many sugar and lipid metabolites. Further, the analysis determined an upregulation of pyruvate decarboxylase 6 (*PDC6*). Interestingly, the flux balance distribution of mutant 102a strain had also showed an increase in the flux of reaction catalyzed by pyruvate decarboxylase (Fig. 1). Thus the upregulation of pyruvate decarboxylase and phosphate pathway genes in the transcriptomic data correlated well with the metabolic flux balance distribution data. As there were no other metabolic genes upregulated transcriptomically, we conclude that the upregulation of acetyl-CoA synthetase, NADP^+^ dependent acetaldehyde dehydrogenase and cytoplasmic NADP^+^ isocitrate dehydrogenase fluxes were controlled metabolically rather than by the transcriptome.

### Flux balance distribution of mutant along with trancriptomic constraints reveals truncation of the TCA cycle and an increased demand of phosphate in the mitochondria of the mutant

**Fig. 3:**
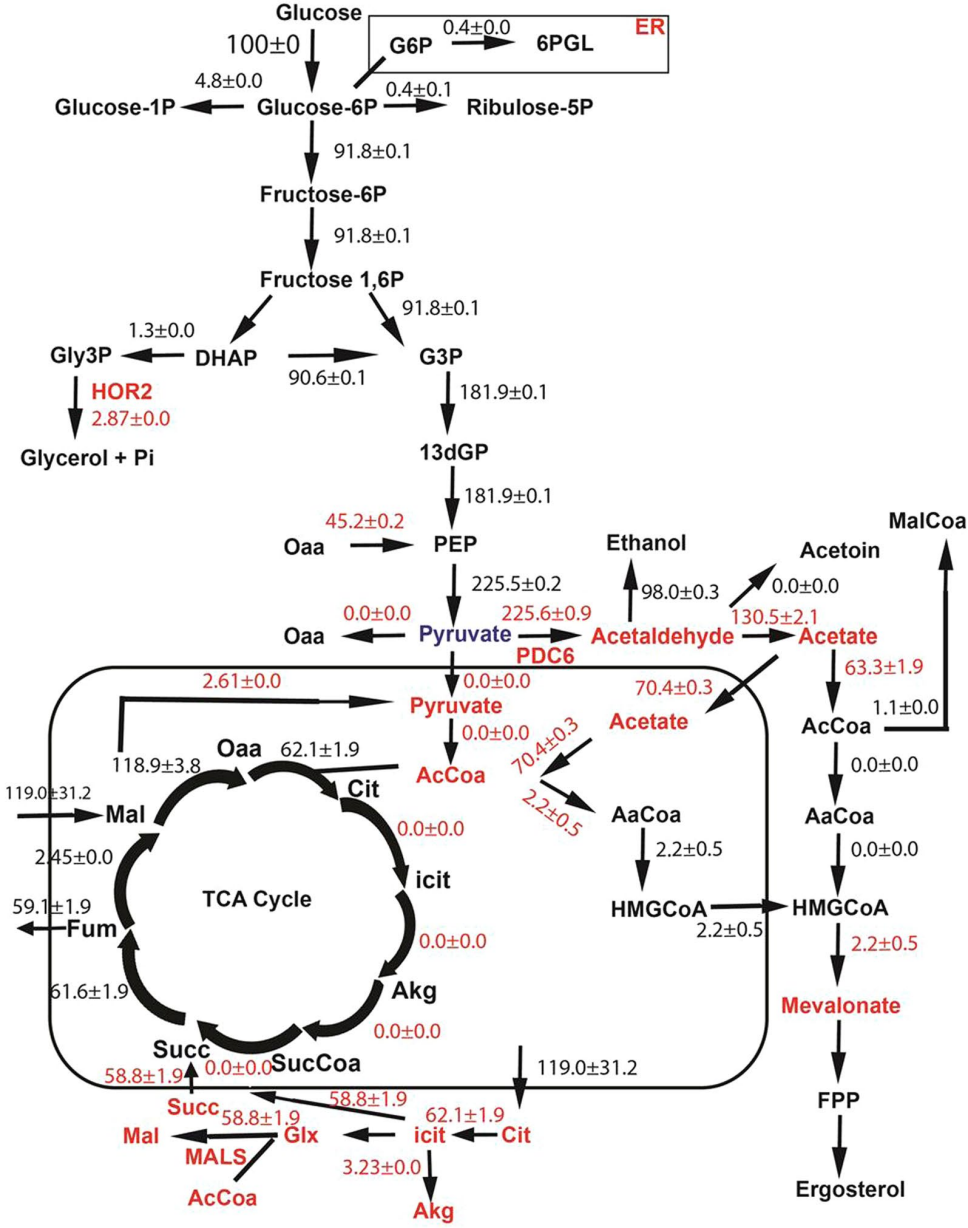
Effect of transcriptomic constraints on the flux distribution in central metabolism of 102a (*spt15_Ala101Thr*) strain. *PDC6*—pyruvate decarboxylase 6, *HOR2*—glycerol-3-phosphate phosphatase, *MALS*—malate synthase

The data from the growth experiments for control 101a and mutant 102a strains were further analyzed by flux balance analysis imposing constraints observed from the transcriptomic analysis and additional diffusion constraints (Additional file 1: Table S3A, S3B and S3C). These constraints imposed a higher flux through the pyruvate decarboxylase reaction (*PDC*), thereby increasing the flux towards acetaldehyde. The mitochondrial acetate was now mainly forced through transfer of cytosolic acetate synthesized from acetaldehyde in the cytoplasm. Thus, the acetate in the mitochondria is channeled to Acetyl-CoA (62/70) and HMG-CoA (2.5/70) thereby increasing the flux through the isoprenoid pathway as shown in Fig. 3. Under these phenotypic conditions, the flux balance predicts a truncated TCA cycle in the mitochondria wherein, the citrate is shunted out to the cytoplasm and reconnects at succinate via the glyoxylate shunt. The ATP demand increased by about two folds therefore created phosphate limitation in the cell. The increased demand for mitochondrial phosphate was met from mitochondrial diphosphates through mitochondrial diphosphatases (91.5%) and by transport of cytosolic phosphate to mitochondria (8.1%). From the percentages, we can conclude that the production of mitochondrial diphosphatases was insufficient and hence necessitated the import of phosphate from the cytosol or from extracellular environment. However, in this strain, mitochondrial diphosphatases produce the major amount of phosphate required in mitochondria thereby resulting in reduction of the transport flux from cytosol by 8.8% when compared to the mutant strain without transcriptomic constraints (Additional file 1: Table S6). Therefore, we can propose that due to this high demand of phosphate, the 102a mutant strain causes activation of the PHO regulon so as to increase uptake of phosphate from the environment and also the release of phosphate from stored polyphosphates in the vacuole.

In *spt15_Ala101Thr* mutant strain, the cytosolic NADPH production increased due to increased flux in the reaction caused by NADP^+^—dependent acetaldehyde dehydrogenase. Interestingly, the increased cytosolic NADPH pools are not only consumed through HMG-CoA reductase but also through aspartate semialdehyde dehydrogenase which utilizes NADPH along with the production of cytosolic phosphate (Additional file 1: Table S9). This flux analysis clearly indicates that the cell could utilize several mechanisms to increase phosphate levels in both mitochondria and cytoplasm to meet the increased phosphate demand.

### Increasing extracellular phosphate concentration leads to increased growth rate and pushes the flux to biomass rather than isoprenoid pathway in *spt15_Ala101Thr* mutant

**Fig. 4:**
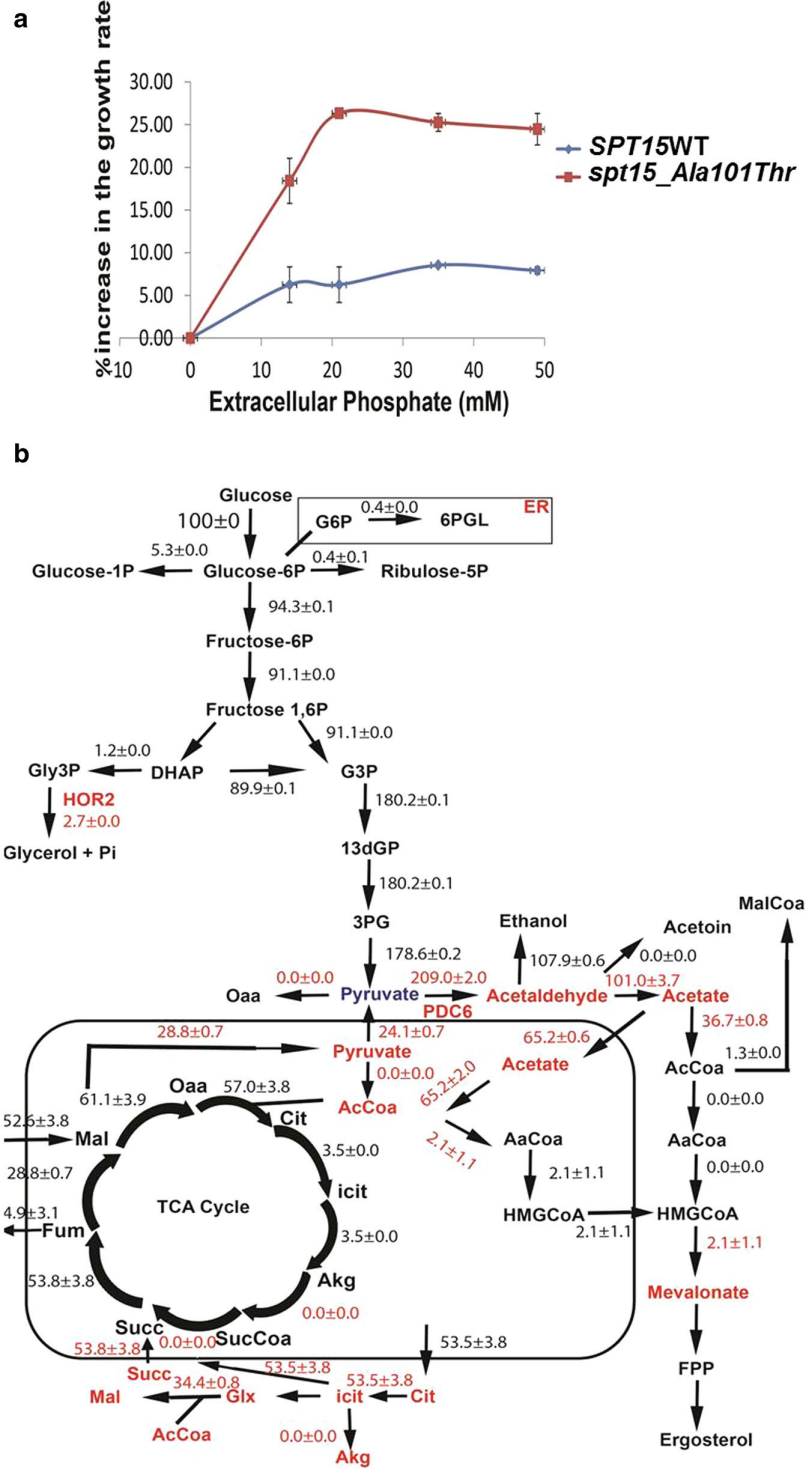
Effect of extracellular phosphate on the growth rate and MVA pathway flux. Effect of different concentration of extracellular KH_2_PO_4_ (**a**) on the growth rate of 101a (*SPT15*WT) and 102a (*spt15_Ala101Thr*) strain (**b**) Effect of extracellular phosphate on the flux distribution in central metabolism of 102a (*spt15_Ala101Thr*) strain with transcriptomic constraints grown with 21 mM concentration of extracellular KH_2_PO_4_

In order to investigate the effect of increasing the extracellular phosphate in the media on growth, control 101a and mutant 102a strains were grown in media containing varying concentrations of phosphate. Both the strains had an increased growth rate with increasing phosphate concentration (Additional file 1: Table S7), saturating at a beyond phosphate concentration of 20 mM (Fig. 4a). However, the mutant strain showed an approximately 25% increase in growth rate while the control strain showed a meagre 5% increase. A greater increase in the growth rate of the mutant strain as compared to control strain suggests that the initial slow growth rate of the mutant was due to limitation of phosphate and thus addition of 21 mM of KH_2_PO_4_ in the media allowed the growth rate to become similar to that of the control strain.

In order to investigate the effect of extracellular phosphate on the isoprenoid pathway flux, growth experiments of control 101a and mutant 102a strains were performed in media with 21 mM concentration of extracellular phosphate. Flux balance distribution of mutant 102a strain was performed with the accumulation rates along with previously mentioned transcriptomic and diffusion constraints (Fig. 4b). With increased phosphate levels, there is a decrease in the flux from pyruvate to cytosolic acetyl-CoA and increase in ethanol formation. Although with the increased phosphate levels, the TCA cycle was truncated with glyoxylate shunt operational in the cytoplasm but it allowed about 3.5% increase in flux towards α-ketoglutarate (α-KG) and decreased flux towards the isoprenoid pathway through HMG-CoA in the mitochondria. Interestingly, the excess pyruvate in mitochondria was transferred to the cytoplasm with a decreased formation of pyruvate through glycolysis (note that the OAA to PEP via phosphoenolpyruvate carboxykinase, ppck is not operational in high phosphate case). Overall, at higher phosphate concentration, the flux balance analysis of mutant 102a strain suggests the increase in TCA cycle flux for favoring increased biomass alongwith a decrease in the isoprenoid pathway flux.

### The role of pyruvate decarboxylase (*PDC6*) towards increasing the isoprenoid pathway flux

**Fig. 5:**
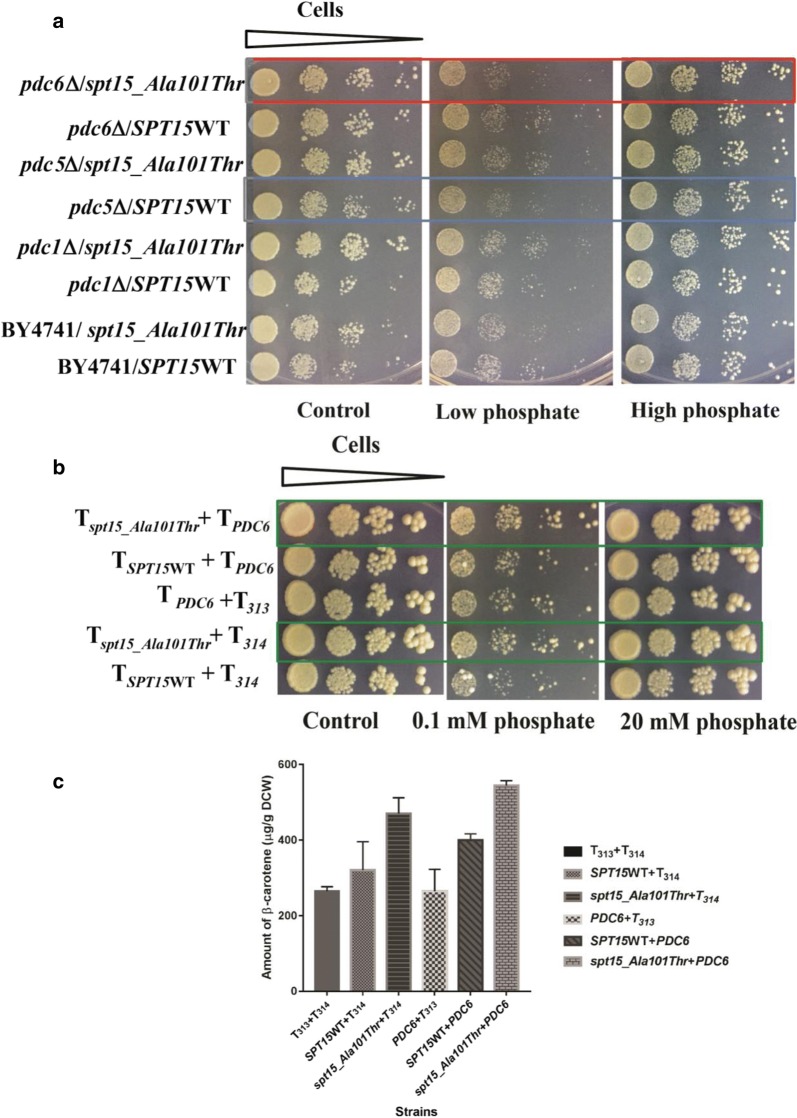
Role of pyruvate decarboxylase (*PDC6*) on pigmentation and growth on 102a (*spt15_Ala101Thr*) mutant strain. **a** Effect of overexpression of *spt15_Ala101Thr* plasmid on the growth of BY4741 (wild type) and *pdc1Δ*, *pdc5Δ* and *pdc6*Δ deletion strains (containing carotenogenic genes (T_PSY1_ and GPD_CRTI_Ala393Thr)_) **b** Effect of overexpression of *PDC6* alone and in combination with either *SPT15*WT or *spt15_Ala101Thr* plasmid on pigmentation of carotenogenic yeast. **c** Amount of carotenoids in the carotenogenic yeast strains overexpressing either *spt15_Ala101Thr* alone or in combination with *PDC6.* In **a**, red box depicts the growth defect observed in *spt15_Ala101Thr* mutant strain in *pdc6Δ* deletion strain under low phosphate conditions and blue box depicts slight growth defect observed in *SPT15*WT strain in *pdc5Δ* deletion strain under low phosphate conditions. In **b**, green boxes depict the two *spt15_Ala101Thr* mutant strains with and without overexpression of *PDC6*, T refers to TEF promoter

It was interesting to note that in the mutant 102a strain, the flux through *PDC* was increased compared to the wild type 101a strain irrespective of whether or not there was excessive phosphate in the media. To investigate the effect on isoprenoid pathway flux, we employed a previously described carotenoid based genetic screen where the genes encoding a phytoene synthase (*Rt*PSY1) and a hyperactive phytoene dehydrogenase (*Rt*CRTI_Ala393Thr) from *R. toruloides* were expressed under strong promoters in *S. cerevisiae*. Increase in pigmentation in this carotenogenic yeast assay strain was demonstrated to reflect an increase in isoprenoid pathway flux [13]. Therefore, in order to investigate the specific role of *PDC6* on the isoprenoid pathway flux in the mutant strain (102a), we compared wild type (BY4741), individual *pdc1*Δ, *pdc5*Δ and *pdc6*Δ deletion strains (Additional file 1: Table S2) using carotenoid based genetic screen. We overexpressed the carotenogenic genes along with either *SPT15*WT (control) or *spt15_Ala101Thr* (mutant) genes. These transformants were grown, serially diluted and spotted under low and high phosphate plates (Fig. 5a). The *pdc6*Δ strain bearing the mutant *spt15_Ala101Thr* plasmid demonstrated a prominent growth defect under low phosphate conditions. However, this defect was not observed under high phosphate conditions (Fig. 5a). Further, *PDC6* was overexpressed alongwith either *SPT15*WT (control) or *spt15_Ala101Thr* (mutant) plasmid in the strain carrying the carotenogenic genes where pigmentation was a measure of the isoprenoid flux. Interestingly, an increased pigmentation was observed alongwith *spt15_Ala101Thr* mutant plasmid under low as well as high phosphate plates, indicating a higher isoprenoid flux by overexpression of *PDC6* irrespective of phosphate conditions (Fig. 5b). However, chemical analysis under normal phosphate conditions (SD minimal media) suggests that the increase is only marginal (Fig. 5c). However, overexpression of *PDC6* with *SPT15*WT did not show an increase in the pigmentation. These results suggest that in the mutant *spt15_Ala101Thr* strain, under low phosphate conditions, *PDC6* is required for both growth and increase in the isoprenoid flux. Under high phosphate conditions, overexpression of *spt15_Ala101Thr* mutant in *pdc6*Δ strain did not have the growth defect seen in low phosphate conditions. This suggested that *PDC6* confers a growth benefit to *spt15_Ala101Thr* mutant only under low phosphate conditions.

## Discussion

**Fig. 6:**
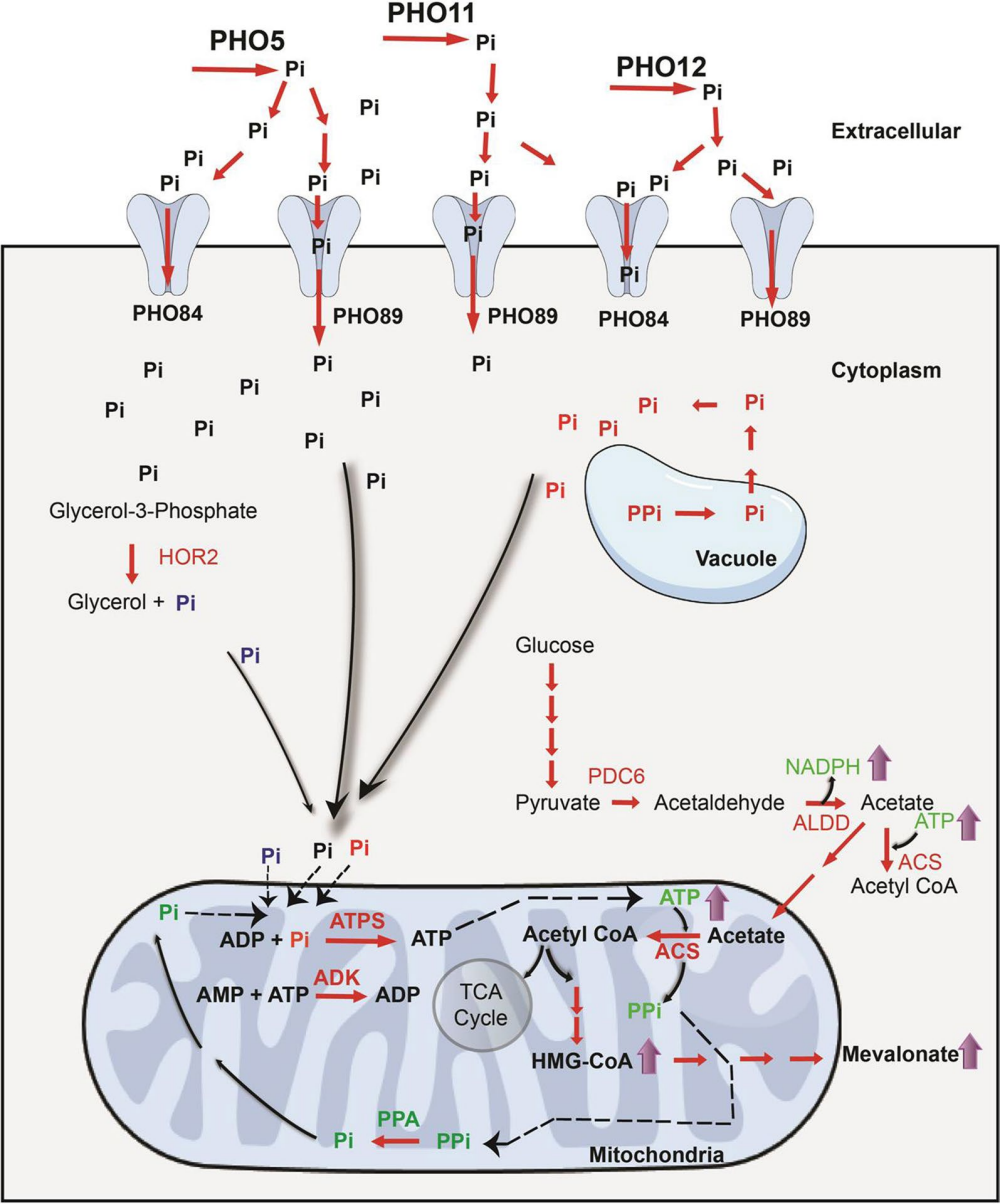
Schematic representation for increase in flux of MVA pathway by 102a (*spt15_Ala101Thr*) mutant strain. *PHO5*- alkaline phosphatase, *PHO11*, *PHO12*- acid phosphatase, *PHO84*, *PHO89*- high affinity phosphate transporters, *PDC6*- pyruvate decarboxylase 6, *HOR2*- glycerol-3-phosphate phosphatase, *ALDD*- acetaldehyde dehydrogenase, *ACS*- acetyl CoA synthetase, *ATPS*- ATP synthase, *ADK*- adenylate kinase

In this study, we have examined the metabolic basis for the increased isoprenoid flux observed in a specific mutant of the TATA binding protein (*SPT15*). The *spt15_Ala101Thr* mutant was isolated in a specific genetic screen designed to identify mutants showing increased isoprenoid flux. The mutant strain demonstrated an increased flux in the isoprenoid pathway along with a requirement of additional phosphate. The limitation in phosphate was also indicated by an upregulation of phosphate transfer genes (*PHO84, PHO89*) as seen from the microarray experiments. The importance of phosphate demands in the *spt15_Ala101Thr* mutant, which was seen from both the flux balance and the microarray data, was interesting in the light of recent observations which have suggested phosphate limitation as being critical for increased flux in both the isoprenoid and lipid pathways [15, 16]. The strategy to limit phosphate in media has also been exploited to increase the yield of amorphadiene [1], where phosphate limitation conditions limited growth but pushed the carbon flux towards production of amorphadiene. Despite these various studies, the exact mechanism of how phosphate limitation leads to these increased yields has never been adequately elucidated. In the present study, the microarray and flux balance analyses also indicated an increased expression of pyruvate decarboxylase (*PDC*) and a corresponding increase in flux from pyruvate to acetaldehyde. Based on these analyses, both in limiting and excess phosphate levels in the medium, an overall mechanism can be hypothesized (Fig. 6).

The pyruvate formed as a product of glycolysis is channeled to acetaldehyde and then to acetate in the cytoplasm in the mutant 102a strain, through overexpression of *PDC6*. Since acetaldehyde dehydrogenases are not transcriptionally upregulated under these conditions, it is likely that the increased flux is through posttranscriptional or posttranslational mechanism [17]. The acetate is later transferred to mitochondria and converted to acetyl-CoA, thereby increasing the demand for ATP. The increased pool of acetyl-CoA was channeled to HMG-CoA which increased the flux towards isoprenoid pathway. The remaining carbon was routed towards TCA cycle, albeit a truncated one. The TCA cycle was completed via the glyoxylate shunt as opposed to via succinyl-CoA since this requires additional phosphate for ATP generation. The glyoxylate shunt balances the CoA pool without the need for additional phosphate and hence is the preferred pathway to complete the TCA cycle, especially under phosphate limitation.

Higher mitochondrial ATP levels were regenerated for production of excess acetyl-CoA from acetate in the mitochondria via ATP synthase, which led to increased phosphate requirements. The increased requirement of mitochondrial phosphate pools was met through multiple routes such as, by increasing uptake of phosphate from extracellular environment, increasing the flux of phosphate producing reactions in the cytosol, importing phosphate from the cytosol to the mitochondria and increasing production of phosphate from diphosphates by diphosphatases in mitochondria. Further, the phosphate was appropriately balanced by not utilizing reactions requiring excess ATP, such as reaction catalyzed by succinate-CoA ligase.

A recent study has shown that for increasing isoprenoid flux, the increased carbon flux to isoprenoid pathway could be achieved through increasing the flux from pyruvate to acetyl-CoA through pyruvate dehydrogenase complex in addition to the increased flux in pyruvate decarboxylase for acetaldehyde [18]. We simulated the above conditions in 102a mutant strain with constraints of the observed growth and accumulation rates, but could not get a feasible solution. This indicated that the pyruvate decarboxylase flux towards increased acetaldehyde was probably the only possible route to increase the isoprenoid flux in the 102a mutant strain.

The observation that the 102a mutant strain essentially requires *PDC6* for growth and increased flux through the isoprenoid pathway under phosphate limitation conditions suggested that, upregulation of *PDC6* is exploited by the mutant strain for increased acetaldehyde, acetate and acetyl-CoA pools (both in cytosol and mitochondria) essential for the increased flux through the isoprenoid pathway. In *S. cerevisiae*, there are three genes coding for pyruvate decarboxylase, *PDC1*, *PDC5* and *PDC6*. *PDC* activity in *S. cerevisiae* is primarily due to expression of *PDC1*. *PDC6* is a minor isoform, where transcription is dependent upon glucose and ethanol and sulfur limited conditions [19]. The mechanism for the up-regulation of *PDC6* under phosphate limitation conditions is unclear and requires detailed investigation. In any case, the possibility that the *PDC* enzymes could be a route for increasing the isoprenoid pathway flux in *S. cerevisiae* appears to be novel and reviewing the literature seems to indicate that this pathway has not been observed so far.

## Conclusions

We conclude that *spt15_Ala101Thr* mutant increases the isoprenoid pathway flux via rewiring of central carbon metabolism. There is rerouting of pyruvate towards production of acetyl-CoA, TCA cycle and cytosolic NADPH levels. Under phosphate limitation conditions in 102a mutant strain, the excess carbon is pushed to the isoprenoid pathway by increased flux through pyruvate decarboxylase 6 (*PDC6*) from acetaldehyde to acetate to mitochondrial acetyl-CoA. This route also led to increased cytosolic NADPH levels which is required for the isoprenoid pathway. Our results suggest that the route followed by *spt15_Ala101Thr* mutant strain can be utilized for increasing the isoprenoid pathway flux.

## Methods

### Strains and media

*Escherichia coli* strain DH5α strain was used as cloning host. *Saccharomyces cerevisiae* strain BY4741 (*MAT a, ura3Δ0, his3Δ1, leu2Δ0 met15Δ0*) was used in this study. Yeast strains were maintained on yeast extract, peptone and dextrose (YPD) media. Synthetic defined media (SD) contained 0.15% (w/v) yeast nitrogen base (YNB) without ammonium sulphate and amino acids and 0.5% (w/v) ammonium sulphate, 2% (w/v) d-glucose and appropriate amino acids (uracil, histidine, methionine, leucine) (at concentration of 80 mg L^−1^) according to auxotrophic requirements of *S. cerevisiae* strain was used.

Inorganic phosphate free synthetic defined media was prepared from Yeast nitrogen base (YNB) without potassium phosphate, ammonium sulphate and amino acids, 0.5% (w/v) ammonium sulphate, 2% (w/v) d-glucose. Appropriate amino acids (uracil, histidine, methionine, leucine) (at concentration of 80 mg L^−1^) according to auxotrophic requirements of *S. cerevisiae* strain was added. 1 M KH_2_PO_4_ was used to make minimal media of defined phosphate concentration.

To investigate the effect of the increased growth rate of the 102a mutant strain (Additional file 1: Table S2) observed at 21 mM concentration of extracellular phosphate on the flux balance distribution, the mutant strain was grown in minimal media containing 21 mM of KH_2_PO_4_ and the accumulation rates of four extracellular metabolites, glucose, glycerol, ethanol and acetate were estimated during the exponential phase of growth curve (Additional file 1: Table S3).

### Culture conditions

Cell concentration was determined by measuring the absorbance at OD_600_ using a spectrophotometer (Perkin elmer UV/VIS Spectrophotometer, USA). From the exponential phase of growth curve of *SPT15*WT and *spt15_Ala101Thr* mutant strains in same media (SD minimal media), dry cell weight (DCW) was determined. Both *SPT15*WT and *spt15_Ala101Thr* mutant strains have DCW as 0.53 g DCWL^−1^. A single colony of yeast strain was used to inoculate 5 mL minimal medium supplemented with appropriate amino acids and grown overnight at 30 °C with shaking at 250 rpm. Secondary culture was inoculated at 0.05 OD_600_ in 50 mL minimal media in 250 mL shake flask supplemented with appropriate amino acids and grown at 30 °C with shaking at 250 rpm.

### Plasmid vectors, cloning of genes and transformation

The yeast centromeric plasmids p416TEF, pRS313TEF and pRS315TEF were used for cloning and expression of genes. pRS416TEF- *Rt*PSY1, pRS315GPD- *Rt*CRTI (A393T), pRS313TEF- *SPT15*WT and pRS313TEF- *spt15_Ala101Thr* have been described previously [13]. The ORF *PDC6* (1692 bp) was PCR amplified from genomic DNA of *S. cerevisiae* BY4741 strain using specific primers (Additional file 1: Table S10) and cloned downstream of TEF promoter using *Bam*HI and *Xho*I site of pRS314TEF. The schematic plasmid maps of pRS416TEF- *Rt*PSY1, pRS315GPD- *Rt*CRTI _Ala393Thr, pRS313TEF- *SPT15*WT, pRS313TEF- *spt15_Ala101Thr* and pRS314TEF- *PDC6* are shown in Figure S3 in Additional file 1. All these constructs were transformed in yeast using a modified lithium acetate method [20].

### Dilution spotting for growth and color visualization

*Saccharomyces cerevisiae* strains carrying different plasmids were grown overnight in minimal media supplemented with appropriate amino acids, re-inoculated in fresh media at 0.1 OD_600_ and grown at 30 °C till 0.6–0.8 OD_600_. Cells were then harvested and washed with deionized water and resuspended in water to make dilutions of different OD_600_ − 0.2, 0.02, 0.002, 0.0002. 10 µL of different dilutions were spotted on SD plates supplemented with appropriate amino acids.

### Microarray analysis: Growth of cells, RNA extraction and analysis

#### Growth of cells

*S. cerevisiae* cells constitutively expressing either *SPT15*WT or *spt15_Ala101Thr* were grown overnight in SD media containing appropriate amino acids and then re-inoculated at 0.1 OD_600_ in same medium. At an OD_600_ − 1.5, the cells were harvested, washed and re-suspended in RNA*later*^*®*^ (10^8^ cells). Four samples [two for *SPT15*WT (control, ABC 101a) and two for (*spt15_Ala101Thr*) (test, ABC 102a)](Additional file 1: Table S2) were used for microarray analysis.

#### RNA Isolation, labelling, hybridization, scanning and data analysis

The protocol for RNA isolation, labeling, hybridization, scanning was followed as described previously [21]. Microarray data was analyzed and submitted to the Gene Expression Omnibus (GEO) database (GSE10859).

### RNA isolation and RT- PCR

RNA isolation and cDNA synthesis was done as described previously [22]. *S. cerevisiae* transformants constitutively over expressing either *SPT15*WT or *spt15_Ala101Thr* were grown in SD media supplemented with appropriate amino acids at 30 °C. When cells reach to an OD_600_ − 1.5, cells were harvested. Total RNA was isolated by hot acid phenol method [22], followed by DNase treatment (cat. no. M6101, Promega) at 30 °C for 15 min. RNA was cleaned up with Zymo Spin II column (Zymo research, cat. no. C1008-250). 3 µg of total RNA was used for cDNA synthesis using reverse transcriptase and random-hexamer primers (GoScript™ Reverse Transcription system (cat. no. A5000) Promega) at 42 °C for 16 h. Real Time quantitative PCR (RT-qPCR) was carried out on LightCycler^®^ 480 II System (Roche molecular Diagnostics) by using SYBR green dye-based reagents (Maxima Sybr Green QPCR Master Mix, cat. no. K0251, Thermofisher).

### Measurement of extracellular metabolites by HPLC

Concentration of extracellular metabolites such as glucose, ethanol, acetate and glycerol in the media during exponential phase of growth of yeast transformants were measured by JASCO HPLC system using Aminex HPX-87H column (Bio-Rad, Hercules, CA, USA) using a previous protocol [23]. Briefly, 1 mL of samples were collected and centrifuged and then supernatant was filtered through 0.2 µm filter before running through the column. The column was kept at 65 °C and 0.01 N sulfuric acid was used as a mobile phase with a flow rate of 0.5 mL min^−1^. All the experiments were performed in duplicates and error bars represent the standard error mean of two biological replicates.

### Simulation studies for flux balance analysis

Constraint-based flux balance analysis (FBA) was performed using the manually curated metabolic model of *Saccharomyces cerevisiae,* iMM904 [18]. The curated model iMM904 is constrained for reversibility of some reactions, inactivation of some reactions and addition of transport reactions for cofactor utilization. The total reactions in this manually curated model is 1581. The model was imported into COBRAPy environment [24, 25] and optimized using the built-in linear programming solver [24]. For each strain (control, mutant), we constrained uptake and secretion rates of the glucose, ethanol, acetate and glycerol metabolites using experimental measurements (Additional file 1: Table S3) and optimized the model by maximizing the biomass as the objective function and validated the predicted growth rates with experimentally measured values. The flux in transaldolase reaction (TALA) is set to lower bound to 11 units normalized to 100 units of glucose [18].

Further, we computed the maximum flux possible in isoprenoid pathway for convergence in the control to be 0.48 units normalized to 100 units of glucose. In the mutant strain, the maximum isoprenoid flux (HMGCOAR), under the given constraints, predicted by the model is 18.8 units normalized to 100 units of glucose. We had previously experimentally determined that the increase in flux in isoprenoid pathway in the mutant strain is approximately 2.25 fold higher than control strain [13]. Therefore, the flux in the isoprenoid pathway (HMGCOAR) was set to 1.5 units normalized to 100 units of glucose. For flux balance distribution of the mutant strain with additional transcriptomic constraints, the constraints were calculated based on flux values of control strain, 101a (Additional file 1: Table S3B). These constraints along with accumulation rates of metabolites and transport/diffusion constraints (Additional file 1: Table S3C) were used to predict fluxes for isoprenoid pathway which gives 2.2 units normalized to 100 units of glucose which is consistent with experimental observation in our previous study. The flux values shown in figures were normalized to 100 units of glucose.

## Additional file

**Additional file 1: Table S1.** Different spt15 variants for phenotypic improvement. **Table S2.** List of strains used in the study. **Table S3.** A. Parameters for simulations of flux balance distribution B. Transcriptomic constraints for flux balance distribution of 102a. C. Transport/Diffusion constraints. **Table S4.** ATP production and consumption normalized to 100 units of glucose. **Table S5.** List of upregulated and downregulated reactions in 102a (mutant) (without transcriptomic changes) in comparison to 101a (control). **Table S6.** Phosphate production and consumption normalized to 100 units of glucose. **Table S7.** Growth rate of 101a and 102a at different concentration of phosphate. **Table S8.** NADH production and consumption normalized to 100 units of glucose. **Table S9.** NADPH production and consumption normalized to 100 units of glucose. **Table S10.** List of oligonucleotides and their sequence used in the study. **Fig. S1.** Growth curve kinetics of *S. cerevisiae* BY4741 strain carrying either control 101a (*SPT15*WT) or mutant 102a plasmid (*spt15_Ala101Thr*). **Fig. S2.** Detailed flux distribution in central metabolism of control 101a (*SPT15*WT) and mutant 102a (*spt15_Ala101Thr*) strain without transcriptomic changes. **Fig. S3.** Schematic representation of plasmid maps (A) pRS416TEF-*Rt*PSY1 (B) pRS315TEF-*Rt*CRTI_Ala393Thr (C) pRS313TEF-*SPT15*WT (D) pRS313TEF-*spt15_Ala101Thr* (E) pRS314TEF-*PDC6*. *Rt*PSY1, *Rt*CRTI_Ala393Thr corresponds to phytoene synthase and hyperactive mutant of phytoene dehydrogenase from *Rhodosporidium toruloides*, *SPT15*WT, *spt15_Ala101Thr* corresponds to TATA binding protein, *SPT15* wild type and mutant of *SPT15* from *Saccharomyces cerevisiae*, *PDC6* corresponds to pyruvate decarboxylase 6 gene from *S. cerevisiae. TEF* promoter- promoter from Translation elongation factor gene, *GPD* promoter- promoter from Glyceraldehyde-3-phosphate dehydrogenase gene, *CYC1* terminator- terminator from cytochrome C gene. *URA3*, *LEU2*, *TRP1* and *HIS3* are auxotrophic markers for yeast, *S. cerevisiae.*

## Abbreviations

T: TEF
AcCoa: acetyl CoA
ICDH: isocitrate dehydrogenase
PDC: pyruvate decarboxylase
MVA: mevalonic acid
