# Supplementary material for: Role of phosphate limitation and pyruvate decarboxylase in rewiring of the metabolic network for increasing flux towards isoprenoid pathway in a TATA binding protein mutant of Saccharomyces cerevisiae
Source: Microb Cell Fact. 2018 Sep 21;17:152. doi: 10.1186/s12934-018-1000-1 (PMC6149198; doi:10.1186/s12934-018-1000-1)
Supplement: Supplementary file 1 — Additional file 1: Table S1. Different spt15 variants for phenotypic improvement. Table S2. List of strains used in the study. Table S3. A. Parameters for simulations of flux balance distribution B. Transcriptomic constraints for flux balance distribution of 102a. C. Transport/Diffusion constraints. Table S4. ATP production and consumption normalized to 100 units of glucose. Table S5. List of upregulated and downregulated reactions in 102a (mutant) (without transcriptomic changes) in comparison to 101a (control). Table S6. Phosphate production and consumption normalized to 100 units of glucose. Table S7. Growth rate of 101a and 102a at different concentration of phosphate. Table S8. NADH production and consumption normalized to 100 units of glucose. Table S9. NADPH production and consumption normalized to 100 units of glucose. Table S10. List of oligonucleotides and their sequence used in the study. Fig. S1. Growth curve kinetics of S. cerevisiae BY4741 strain carrying either control 101a (SPT15WT) or mutant 102a plasmid (spt15_Ala101Thr). Fig. S2. Detailed flux distribution in central metabolism of control 101a (SPT15WT) and mutant 102a (spt15_Ala101Thr) strain without transcriptomic changes. Fig. S3. Schematic representation of plasmid maps (A) pRS416TEF-RtPSY1 (B) pRS315TEF-RtCRTI_Ala393Thr (C) pRS313TEF-SPT15WT (D) pRS313TEF-spt15_Ala101Thr (E) pRS314TEF-PDC6. RtPSY1, RtCRTI_Ala393Thr corresponds to phytoene synthase and hyperactive mutant of phytoene dehydrogenase from Rhodosporidium toruloides, SPT15WT, spt15_Ala101Thr corresponds to TATA binding protein, SPT15 wild type and mutant of SPT15 from Saccharomyces cerevisiae, PDC6 corresponds to pyruvate decarboxylase 6 gene from S. cerevisiae. TEF promoter- promoter from Translation elongation factor gene, GPD promoter- promoter from Glyceraldehyde-3-phosphate dehydrogenase gene, CYC1 terminator- terminator from cytochrome C gene. URA3, LEU2, TRP1 and HIS3 are auxotrophic markers for yeast, S. cerevisiae. [file 12934_2018_1000_MOESM1_ESM.pdf]

Role of phosphate limitation and pyruvate decarboxylase in rewiring of the metabolic network for increasing flux towards isoprenoid pathway in a TATA binding protein mutant of *Saccharomyeces cerevisiae*

Manisha Wadhwa[1], Sumana Srinivasan[2], Anand K Bachhawat[1*], K. V. Venkatesh[2*]

[1]Department of Biological Sciences, Indian Institute of Science Education and Research, Mohali., India

[2]Department of Chemical Engineering, Indian Institute of Technology, Bombay., Mumbai, India.

Authors: manishawadhwatalla@gmail.com, sumanas@iitb.ac.in

*Corresponding authors: anand@iisermohali.ac.in, venks@iitb.ac.in

Tel : +91 172 2293154, +91 222 5767223

Table S1. Different spt15 variants for phenotypic improvement

**spt15 variants**	**Phenotype**	**Group**	**Residues**	**Location**	**Effect on growth rate**
Spt15-300	Glucose and Ethanol tolerance	Hal Alper et al., 2006	Phe177Ser (F177S)<br>Tyr195His (Y195H)<br>Lys218Arg (K218R)	Second repeat element of conserved C-terminal domain	High growth rate of mutant compared to wild type
Spt15-25	Xylose utilization and tolerance	Liu et al., 2008	-	-	High growth rate of mutant compared to wild type
Spt15-M1<br>Spt15-M2<br>Spt15-M3<br>Spt15-M4<br>Spt15-M5	Ethanol tolerance	Yang et al., 2011	M1- K201N, G216S, N225Stop<br>M2-L76V,L175S<br>M3-S42N, C78R,S163P,I212N<br>M4-K15T,W26C<br>M5-G192D	N-terminal domain and repeat element 1 and 2 of conserved C-terminal domain	Not checked
M1, M2, M3	Increased isoprenoid flux	Wadhwa and Bachhawat., 2016	spt15R98H<br>spt15A100V<br>spt15A101T	N stirrup region of C-terminal core domain	Low growth rate

Table S2. List of strains used in the study

**Strain Name**	**Genotype**	**Reference**
BY4741	*MAT a, ura3Δ0, his3Δ1, leu2Δ0 met15Δ0*	Euroscarf
ABC 101a	BY4741 pRS313TEF-*SPT15*WT	This Study
ABC 102a	BY4741 pRS313TEF-*spt15_Ala101Thr*	This Study
ABC 5714 (*pdc1*Δ)	BY4741*; MAT a, ura3Δ0, his3Δ1,leu2Δ0, met15Δ0, YLR044c::kan MX4*	Euroscarf
ABC 5716 (*pdc5*Δ)	BY4741*; MAT a, ura3Δ0, his3Δ1,leu2Δ0, met15Δ0, YLR134w::kan MX4*	Euroscarf
ABC 5722 (*pdc6*Δ)	BY4741*; MAT a, ura3Δ0, his3Δ1,leu2Δ0, met15Δ0, YGR087c::kan MX4*	Euroscarf

Table S3. A. Parameters for simulations of flux balance distribution

**Specific rate in exponential phase** (mmol/gDCW/h)	**101a (Control)**	**102a (mutant)**	**102a (mutant) (at 21 mM Phosphate)**
Glucose consumption	8.35±0.17	6.65±0.05	7.18±0.07
Ethanol secretion	9.20±0.16	6.52±0.04	7.75±0.03
Glycerol secretion	0.106±0.00	0.08±0.00	0.08±0.00
Acetate secretion	0.00±0.00	0.00±0.00	0.00±0.00

B. Transcriptomic constraints for flux balance distribution of 102a

To examine more rigorously the consequence of transcriptomic changes (from microarray data) on flux balance distribution of mutant 102a strain, transcriptomic constraints for three up-regulated genes glutamate decarboxylase (*GAD1*), pyruvate decarboxylase (*PDC6*) and glycerol-3-phosphatase (*HOR2*)) and two down-regulated genes (methyl citrate dehydratase (*PDH1*) and imidazole glycerol phosphate dehydratase (*HIS3*)) from microarray data were calculated based on the simulated flux values from flux balance distribution of wild type 101a strain

**Gene/ID**	**Flux in 101a (control)**	**Transcriptomic fold change in 102a (mutant)**	**102a (mutant) (Upper Bound)**	**102a (mutant) (Lower Bound)**
GLUDC	0	2.15	0.0215	0.0215
PYRDC	12.95	2.1	27.2	15
G3PT	0.106	1.8	0.191	0.191
MCITDm	0.068	0.56	0.068	0.038

IGPDH	0.015	0.42	0.015	0.0063

GLUDC- Glutamate decarboxylase, PYRDC- pyruvate decarboxylase,G3PT- glycerol-3-phosphatase, MCITDm- methyl citrate dehydratase, IGPDH- imidazole glycerol-3-phosphatase

C. Transport/Diffusion constraints

In addition to these transcriptomic constraints, additional transport/ diffusion constraints from flux balance distribution of 102a mutant strain (having theoretical maximum mevalonate flux) were introduced at given conditions of growth rate and accumulation rates to simulate flux balance distribution. These constraints were mainly on transport of molecules by diffusion which was not possible to detect in the microarray data. These were transport of acetate (Actm), transport of glucose-6-phosphate in endoplasmic reticulum by diffusion (G6Pter) and glucose-6-phosphate dehydrogenase (endoplasmic reticulum) (G6PDH2er)

**Gene/ID**	**Lower bound flux**
Actm	4.68
G6PDH2er	0.025
G6Pter	0.025

Actm- acetate transport, G6PDH2er- Glucose-6-phosphate dehydrogenase in endoplasmic reticulum, G6Pter- transport of Glucose-6-phosphate to endoplasmic reticulum by diffusion

Table S4. ATP production and consumption normalized to 100 units of glucose

**ATP production**	**101a (control)**	**102a (mutant)**	**102a (mutant) With transcriptomic constraints**	**102a (mutant) With transcriptomic constraints (at 21 mM Phosphate)**
Cytosol	84%	87%	51.3%	51%
Mitochondria	16%	13%	48.7%	49%
Total ATP production (units)	421.3	433.2	794	703
**Mitochondrial ATP consumption**				
Cytosol	99.3%	71%	63.6%	50%
Mitochondria	0.3%	29.2%	36.4%	50%
**Flux in Mitochondrial ATP producing reactions (units)**				
ATP synthase	65.5	74	386.5	344
Succinate CoA ligase	1.43	1.28	-	-
**Flux in Mitochondrial ATP consuming reactions (units)**				
ADP-ATP transporter	66.47	53.23	246.6	174.1
Acetyl CoA synthetase	-	10.52	70.4	85.1
Adenylate kinase	-	10.52	70.4	85.1
Acetylglutmate kinase	0.46	0.93	0.5	0.5

Table S5. List of up regulated and down regulated reactions in 102a (mutant)(without transcriptomic changes) in comparison to 101a (wild type)

**Sr. no.**	**ID**	**Name**	**Fold change in flux**	**Comments**
1	PPA	Inorganic diphosphatase (cytoplasm)	>50 fold	No flux in control strain
2	ALDD2y	Aldehyde dehydrogenase acetaldehyde__NADP	>50 fold	No flux in control strain
3	ACSm	Acetyl CoA Synthetase (mitochondrial)	>50 fold	No flux in control strain
4	ADK1m	Adenylate kinase mitochondrial	>50 fold	No flux in control strain
5	ICDHy	Isocitrate dehydrogenase_ NADP	11.6	No flux in control strain
6	CITtbm	Citrate transport mitochondrial	7.5	No flux in control strain
7.	SUCFUMtm	Succinate Fumarate transport mitochondrial	1.5	No flux in control strain
8.	Actm	Acetate transporter mitochondrial	7.1	
9.	SQLS	Squalene Synthase	3.1	
10.	G6PDH2er	Glucose 6 phosphate dehydrogenase (ER)	3.1	
11.	G6Pter	Glucose 6 phosphate transport (ER)	3.1	
12.	PIt2m	Phosphate transporter mitochondrial	1.2	

13.	NADH2_u6cm	NADH dehydrogenase cytosolic mitochondrial	1.2	
14.	ATPS3m	ATP synthase mitochondrial	1.1	
15.	PYRDC	Pyruvate decarboxylase	1.1	
16.	PYRt2m	Pyruvate transport mitochondrial	0.71	
17.	FUMm	Fumarase mitochondrial	0.5	
18.	PPAm	Inorganic diphosphatase	0.1	
19.	CSm	Citrate synthase	0.1	
20.	AKGMALm	R_alpha_ketoglutarate Citrate_transporter	0	No flux in mutant
21.	ICDHxm	R_Isocitrate_dehydrogenase__NAD_	0	No flux in mutant
22.	PDHm	R_pyruvate_dehydrogenase	0	No flux in mutant

Table S6. Phosphate production and consumption normalized to 100 units of glucose

**Phosphate production**	**101a (control)**	**102a (mutant)**	**102a (mutant) With transcriptomic constraints**	**102a (mutant) With transcriptomic constraints (at 21 mM Phosphate)**
Cytosol	90%	91%	39%	51%
Mitochondria	9.6%	9%	61%	49%
Total Pi production (units)	245.5	255.7	584.3	553.5
**Consumption of cytosolic phosphate**				
Cytoplasm	80.4%	77.5%	86.3%	63.8%
Transport to mitochondria by transporter	19.6%	22.5%	13.8%	36.2%
**Consumption of phosphate in mitochondria**				
ATP synthase	96%	98%	100%	92.4%
Succinyl CoA ligase	2%	2%	-	-
Succinate transporter	2%	-	-	7.7%

Table S7. Growth rate of 101a and 102a at different concentration of phosphate

**Conc. of Extracellular phosphate (mM)**	**Growth rate of 101a (control) (hr^{-1})**	**Growth rate of 102a (mutant) (hr^{-1})**
SD + 0	0.24±0.000	0.19±0.001
SD + 14	0.25±0.006	0.22±0.002
SD + 21	0.26±0.001	0.24±0.001
SD + 35	0.26±0.001	0.24±0.002
SD + 49	0.26±0.001	0.24±0.004

Table S8. NADH production and consumption normalized to 100 units of glucose

**NADH production**	**101a (control)**	**102a (mutant)**	**102a (mutant) With transcriptomic constraints**	**102a (mutant) With transcriptomic constraints (at 21 mM Phosphate)**
Cytosol	91.3%	97.9%	68.2%	72.4%
Mitochondria	8.7%	2.1%	31.8%	27.6%
Total NADH production (units)	199.9	188.7	404.8	374.9
**Cytosolic NADH consumption**				
Ethanol production	60.5%	53.1%	35.4%	39.7%
Acetoin production	12.4%	15.3%	0%	0%
**Mitochondrial NADH production**				
Pyruvate dehydrogenase	51.3%	0%	0%	0%
Malate dehydrogenase	25.9%	46%	92.2%	59%
Isocitrate dehydrogenase	9.9%	0%	0%	0.6%

Table S9. NADPH production and consumption normalized to 100 units of glucose

**NADPH production**	**101a (Control)**	**102a (mutant)**	**102a (mutant) With transcriptomic constraints**	**102a (mutant) With transcriptomic constraints (at 21 mM Phosphate)**
Cytosol	90.3%	88.8%	94.4%	82.4%
Mitochondria	9.7%	11.2%	5.6%	17.6%
Total NADPH production (units)	27.1	27	46.6	16.4
**Cytosolic NADPH production**				
Acetaldehyde dehydrogenase	-	49.4%	91%	89.1
Isocitrate dehydrogenase	-	41.4%	7.3%	-
PPP pathway	87.6%	4.84%	1.7%	2.63%
**Cytosolic NADPH consumption**				
Glutamate dehydrogenase	58.8%	58.9%	40.6%	-
HMG CoA reductase	4%	12.6%	10.1%	31.8%
Asparatate semialdehyde dehydrogenase	5.2%	5.2%	15.2%	10.1%

PPP- Pentose phosphate pathway

Table S10. List of oligonucleotides and their sequence used in the study

Oligomer	Sequence 5'-3'
PDC6 BamHI FP	ATGCGGGATCCATGTCTGAAATTACTCTTGGAAAATAC
PDC6 XhoI RP	ATGCTCTCGAGTTATTGTTTGGCATTTGTAGCGG
PHO5 FP RT	CTCGTGATTTGCCTGAAGGTTG
PHO5 RP RT	CCATTTCCAAATCATCGTCAT
PHO84 FP RT	GGCAACAAGTTAAGACCATCTC
PHO84 RP RT	CAACAATATCAGCTAAAGTACC
PHO89 FP RT	CTTCTAGATCTCTAAAATACTG
PHO89 RP RT	GCAGTAGCAAATGTTAACCAAC

Restriction sites are underlined

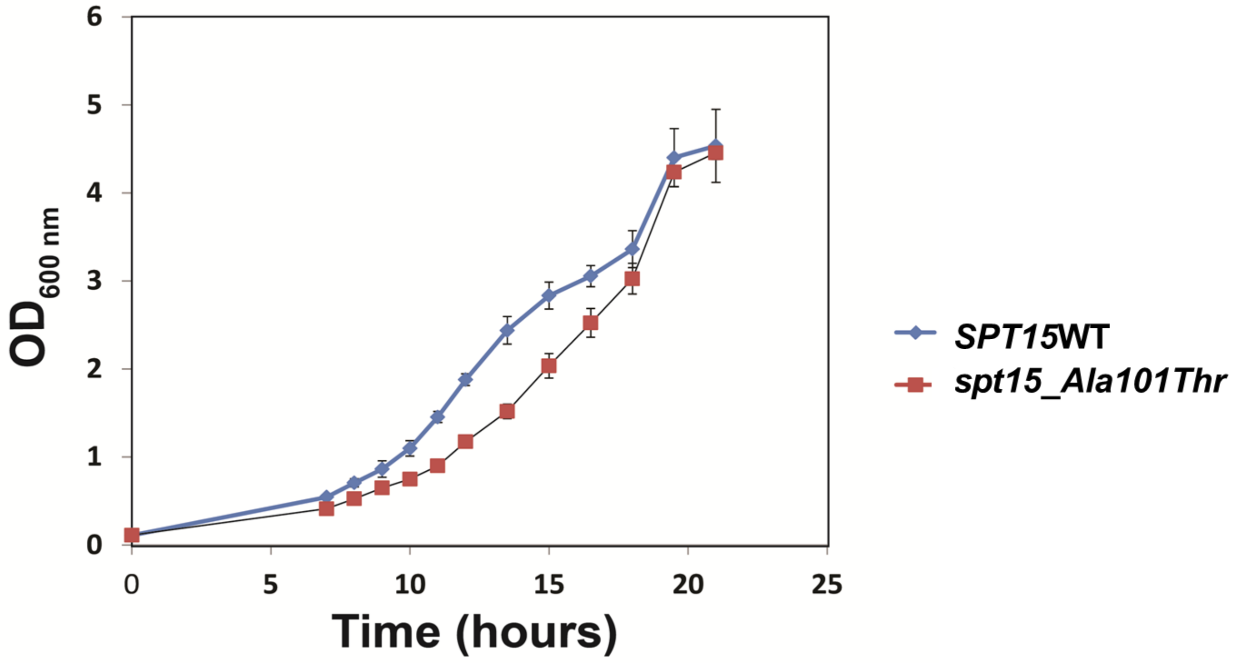

Fig. S1 Growth curve kinetics of *S. cerevisiae* BY4741 strain carrying either control 101a (*SPT15*WT) or mutant 102a plasmid (*spt15_Ala101Thr*).

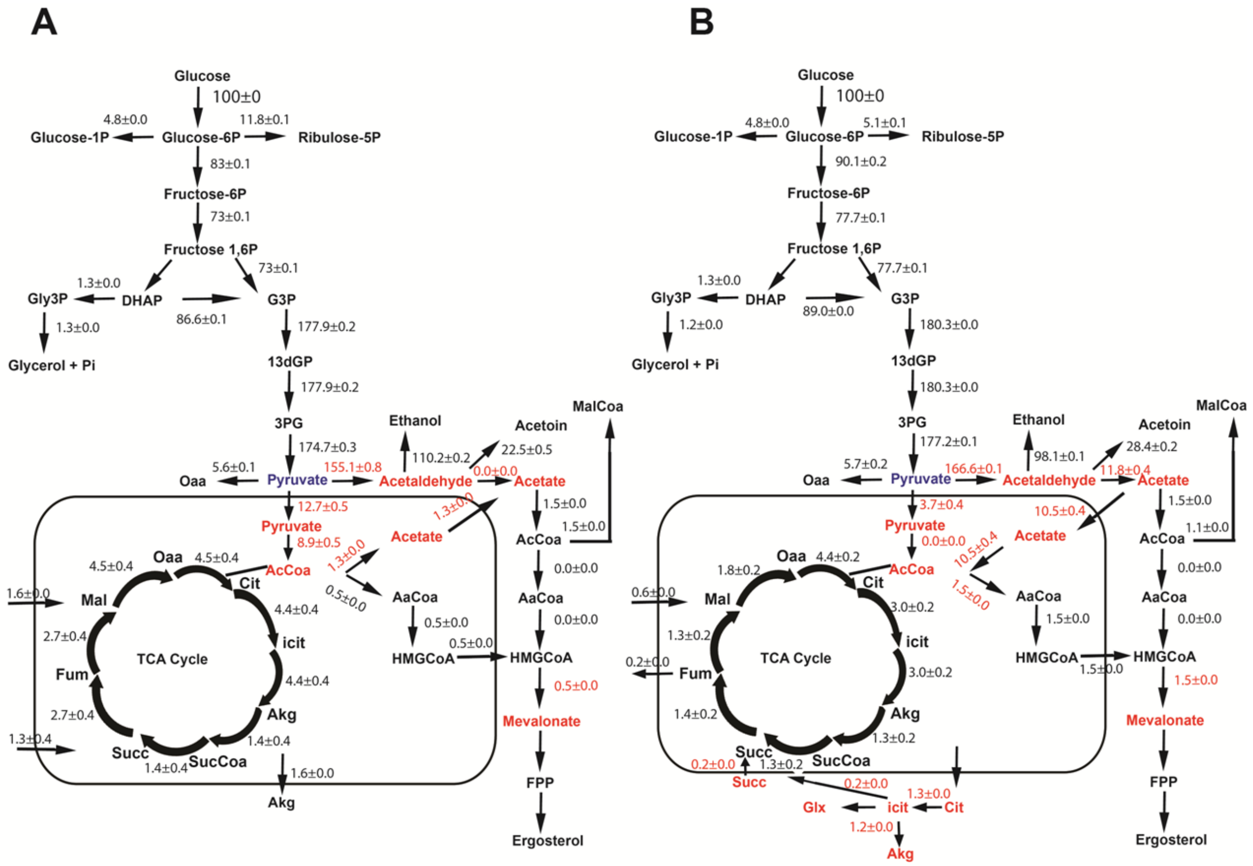

Fig. S2 Detailed Flux distribution in central metabolism of (A) control 101a (*SPT15*WT) and (B) mutant 102a (*spt15_Ala101Thr*) strain without transcriptomic changes. The differences in flux distribution between 101a and 102a strain are shown in red color.

A

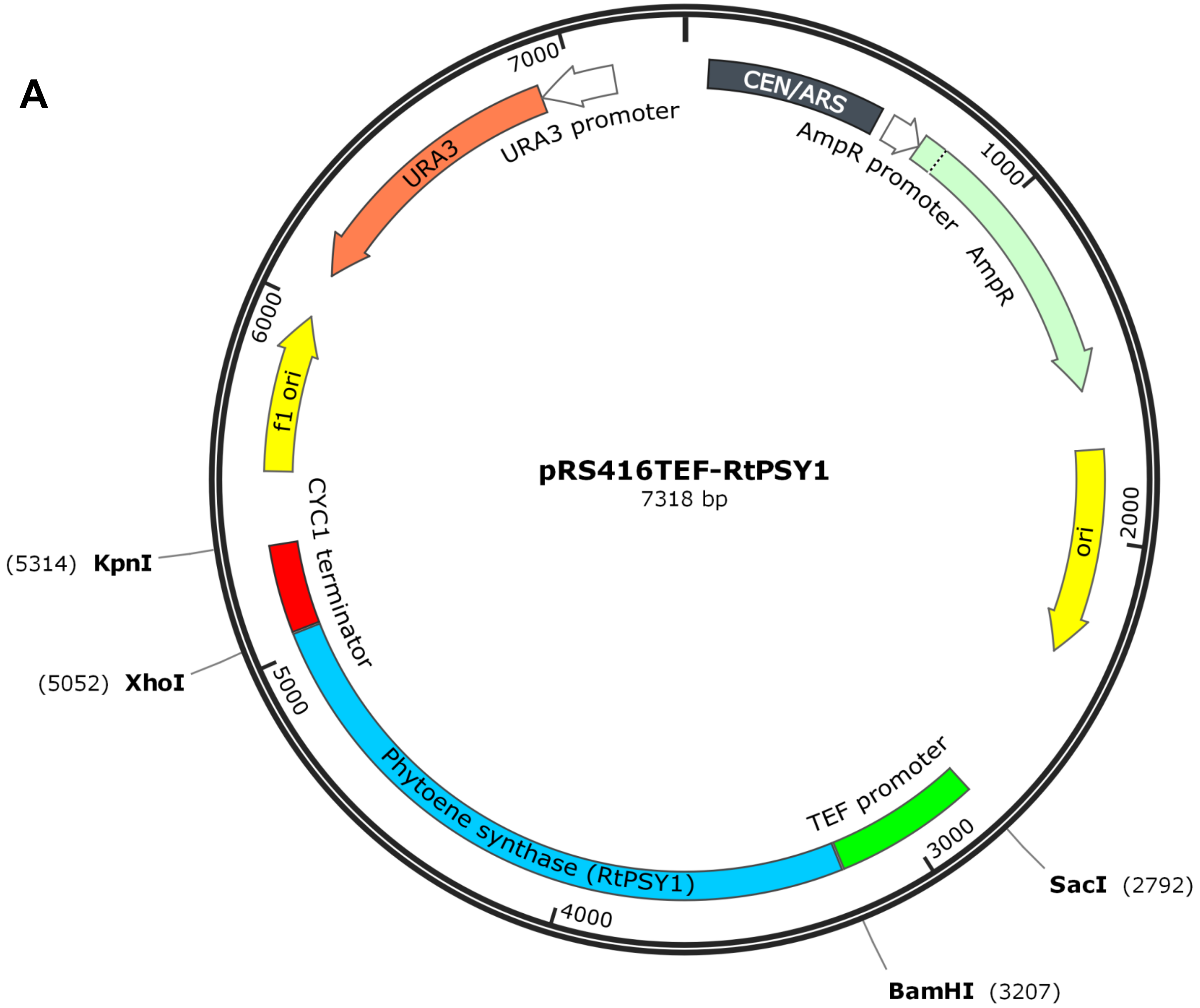

pRS416TEF-RtPSY1
7318 bp
CEN/ARS
AmpR promoter
AmpR
1000
ori
2000
SacI (2792)
3000
BamHI (3207)
TEF promoter
Phytoene synthase (RtPSY1)
4000
5000
(5052) XhoI
(5314) KpnI
CYC1 terminator
f1 ori
6000
URA3
URA3 promoter
7000

B

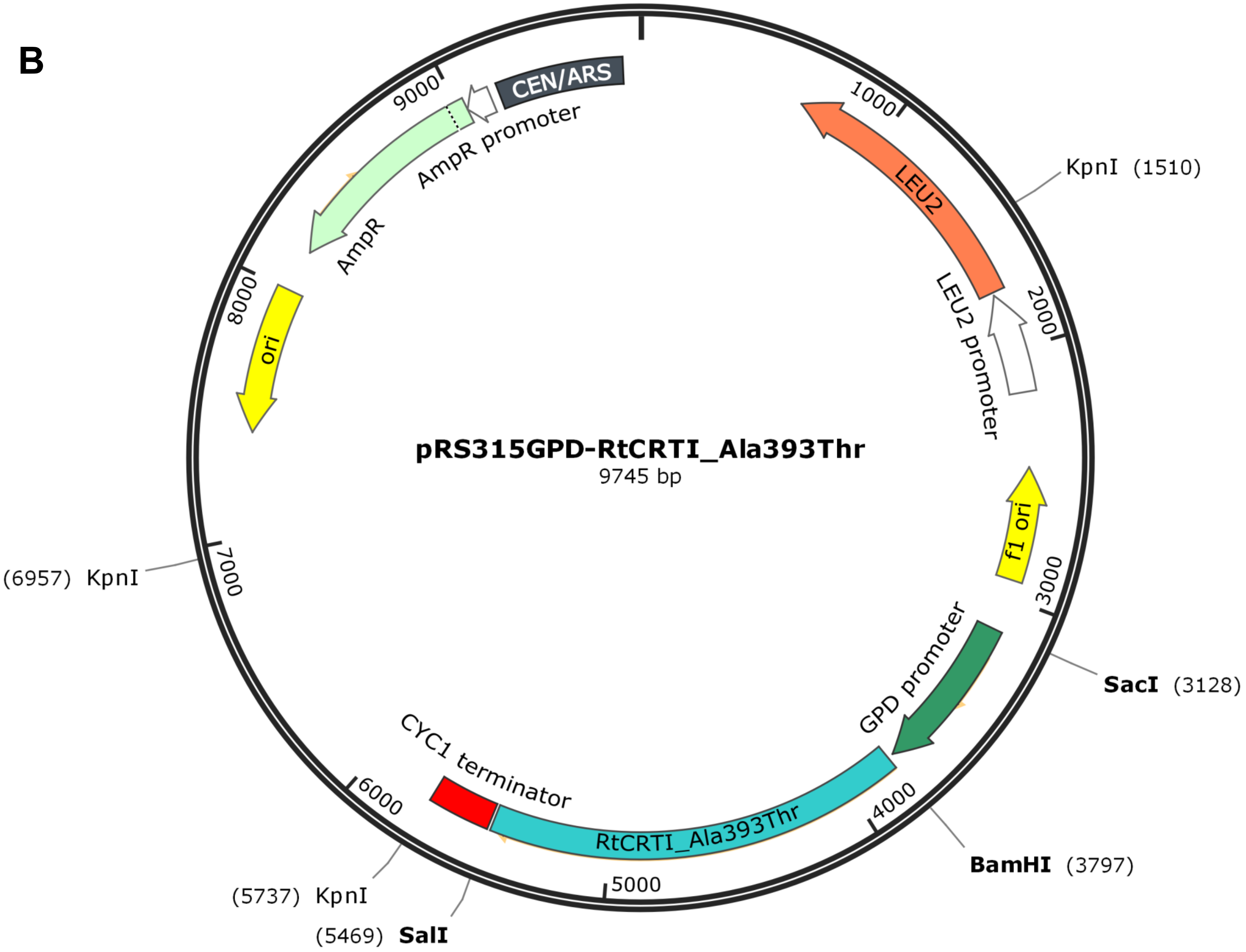
CEN/ARS
AmpR promoter
AmpR
ori
LEU2
LEU2 promoter
f1 ori
GPD promoter
RtCRTI_Ala393Thr
CYC1 terminator
pRS315GPD-RtCRTI_Ala393Thr
9745 bp
KpnI (1510)
SacI (3128)
BamHI (3797)
(5469) SalI
(5737) KpnI
(6957) KpnI
1000
2000
3000
4000
5000
6000
7000
8000
9000

C

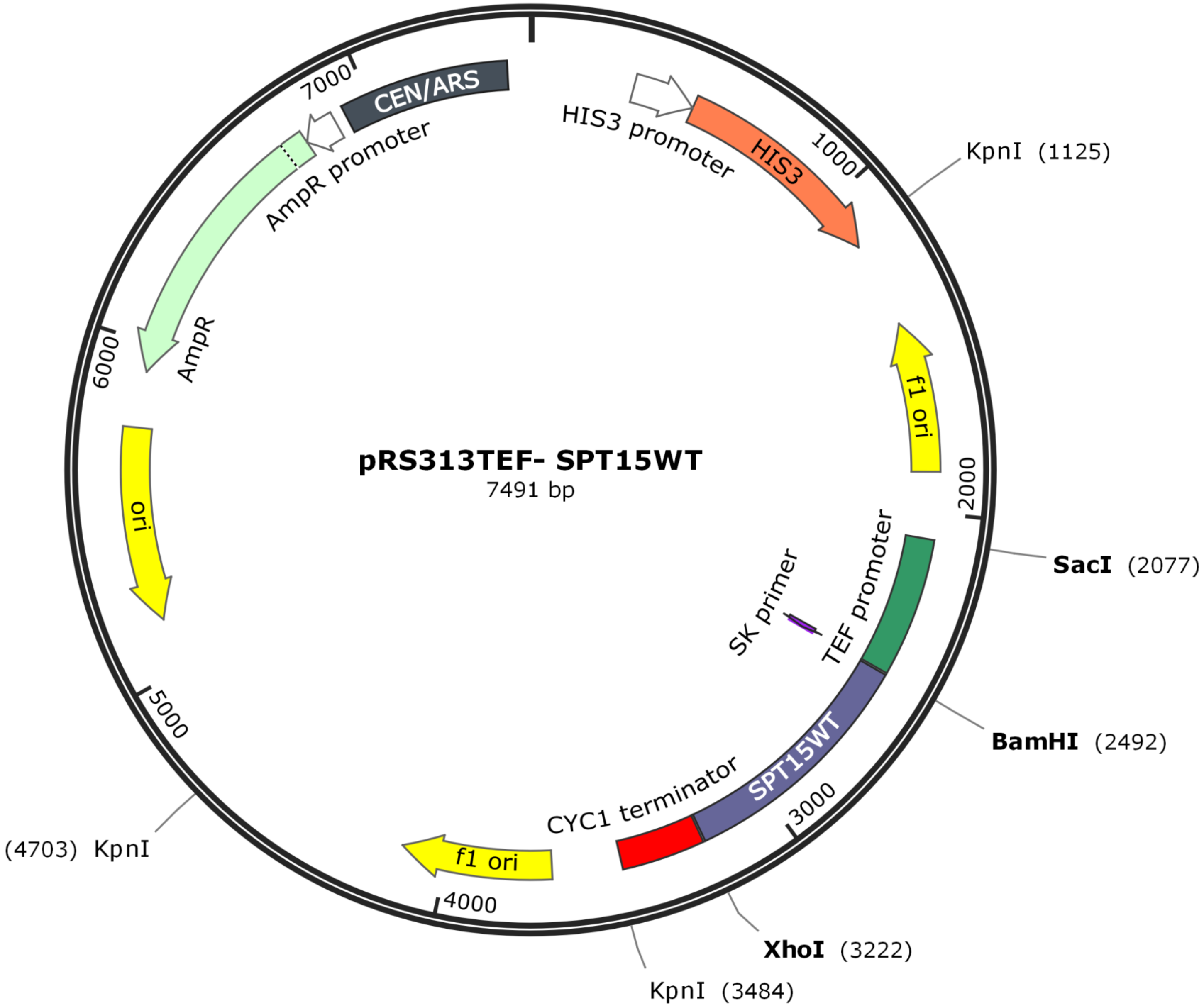
pRS313TEF- SPT15WT
7491 bp
CEN/ARS
HIS3 promoter
HIS3
1000
KpnI (1125)
f1 ori
2000
SacI (2077)
TEF promoter
SK primer
BamHI (2492)
SPT15WT
3000
CYC1 terminator
XhoI (3222)
KpnI (3484)
f1 ori
4000
(4703) KpnI
5000
ori
6000
AmpR
AmpR promoter
7000

D

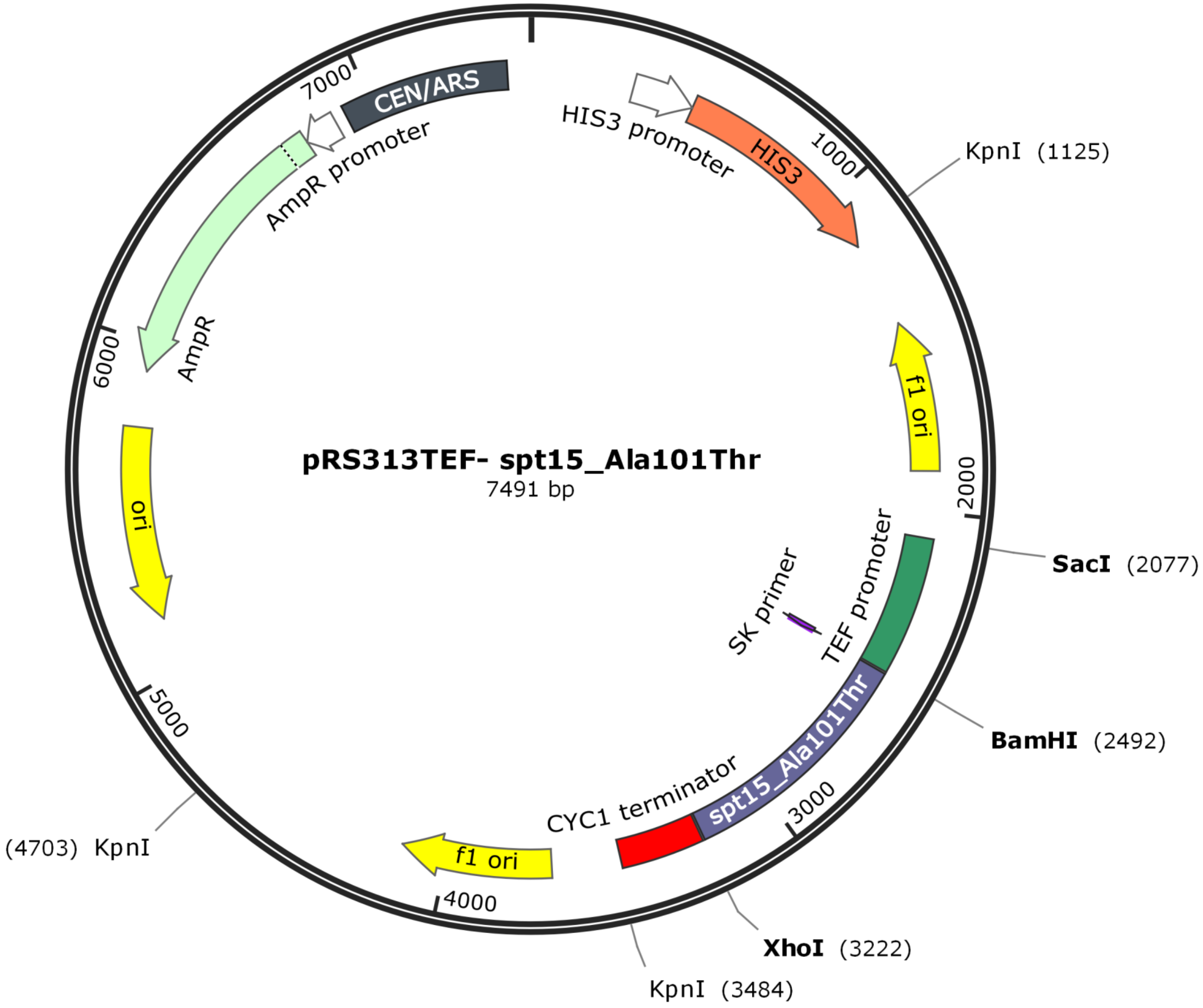
pRS313TEF- spt15_Ala101Thr
7491 bp
CEN/ARS
7000
AmpR promoter
AmpR
6000
ori
5000
(4703) KpnI
4000
f1 ori
CYC1 terminator
spt15_Ala101Thr
3000
KpnI (3484)
XhoI (3222)
BamHI (2492)
SK primer
TEF promoter
SacI (2077)
2000
f1 ori
KpnI (1125)
1000
HIS3
HIS3 promoter

E

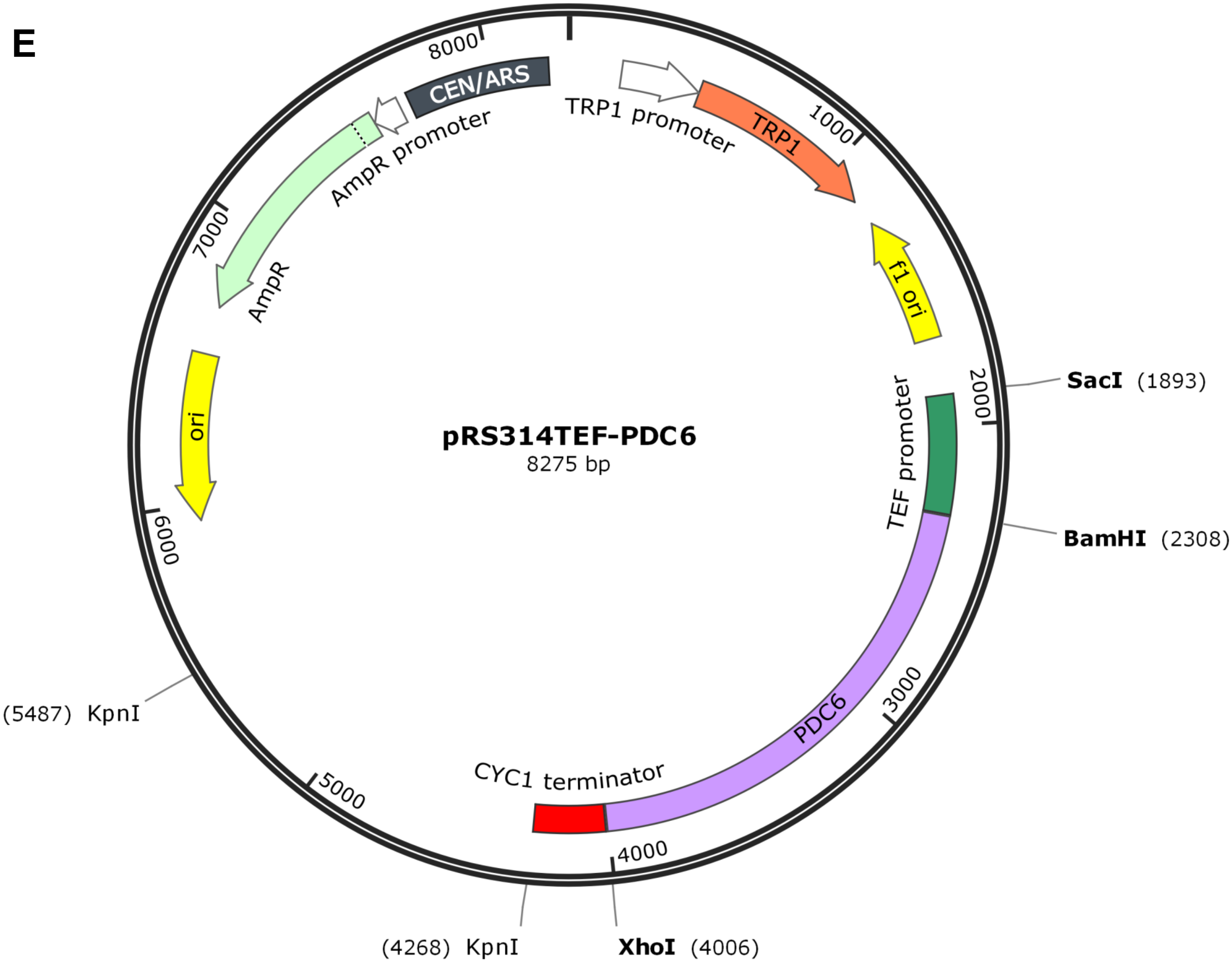

Fig. S3 Schematic representation of plasmid maps (A) pRS416TEF-*Rt*PSY1 (B) pRS315TEF-*Rt*CRTI_Ala393Thr (C) pRS313TEF-*SPT15*WT (D) pRS313TEF-*spt15_Ala101Thr* (E) pRS314TEF-*PDC6*. *Rt*PSY1, *Rt*CRTI_Ala393Thr corresponds to phytoene synthase and mutant of phytoene dehydrogenase from *Rhodosporidium toruloides*, *SPT15*WT, *spt15_Ala101Thr* corresponds to TATA binding protein, *SPT15* wild type and mutant of *SPT15* from *Saccharomyces cerevisiae*, *PDC6* corresponds to pyruvate decarboxylase 6 gene from *S.*

cerevisiae. *TEF* promoter- promoter from Translation elongation factor gene, *GPD* promoter- promoter from Glyceraldehyde-3-phosphate dehydrogenase gene, *CYC1* terminator- terminator from cytochrome C gene. *URA3*, *LEU2*, *TRP1* and *HIS3* are auxotrophic markers for yeast, *S. cerevisiae*.
